# Resorption characteristics of an open architecture biocomposite interference screw after ACL reconstruction

**DOI:** 10.1002/jeo2.70016

**Published:** 2024-10-21

**Authors:** Martin Lind, Torsten Grønbech Nielsen, Flemming K. Nielsen, Ole G. Sørensen, Bjarne Mygind‐Klavsen, Peter Faunø

**Affiliations:** ^1^ Department of Orthopaedics Aarhus University Hospital Aarhus Denmark

**Keywords:** ACL reconstruction, bone in growth, graft fixation, interference screw, resorbable screw

## Abstract

**Purpose:**

Absorbable interference screws for anterior cruciate ligament reconstruction (ACLR) can lead to tunnel widening and cyst formation. The Biosure Regenesorb interference screw (Smith & Nephew). has been developed with an optimised calcium phosphate/polymer composition to promote bone formation during resorption. The present study aims to investigate screw resorption, new bone formation, and tunnel geometry following ACLR with Biosure Regenesorb screw tibial fixation.

**Methods:**

The study is a prospective single‐centre case series of 12 patients with ACL lesions and treated with ACLR using hamstring tendon autograft with Biosure Regenesorb interference screw tibial fixation with a two‐year follow‐up period. The Biosure Regenesorb consists of 65% polylactide‐glycolic acid poly. 20% calcium sulphate and 15% tricalcium phosphate. Primary endpoint: Tunnel volume. implant volume and new bone formation in the tibial tunnel is evaluated by quantitative computed tomography (CT) scanning. Secondary endpoints: Arthrometric knee laxity, International Knee Documentation Committee (IKDC) and Knee Osteoarthritis and Injury Outcome Scores (KOOS) and Tegner activity scale.

**Results:**

Screw volume decreased to 44% within the two‐year follow‐up period while tunnel volume remained unchanged. Only a minor amount (<1% of tunnel volume) of new bone formation in the screw remnants was observed. Sagittal knee laxity at one year was 0.9 mm. The IKDC score increased by 15 points and the KOOS sport and KOOS quality of life scores increased by 25 and 26 points. respectively.

**Conclusion:**

ACLR using Biosure Regenesorb interference screw does not result in tunnel widening. showing a screw resorption of 44% after two years and minor new bone formation. Knee stability and subjective outcome improvements are as expected after other ACLR methods.

**Level of evidence:**

IV.

**Abbreviations**
ACLR: anterior cruciate ligament reconstruction
BTB: bone–tendon–bone graft
CT: computer tomography
HA‐PLLA: hydroxyapatite‐polylactide
IKDC: International Knee Documentation Committee
KOOS: Knee Osteoarthritis and Injury Outcome Score
MRI: magnetic resonance image
PLGA: polylactide‐glycolic acid
TCP: tricalcium phosphate

## INTRODUCTION

The long‐term success of an anterior cruciate ligament reconstruction (ACLR) depends on the graft's ability to heal in the bone tunnel [9].

Fixation of ACL grafts in bone tunnels is typically accomplished using interference screws. Interference screws may be composed of various materials. including metallic. absorbable ceramics and inert polymers. Bioabsorbable materials for interference screws may offer advantages over metallic screws. including reduced risk of graft injury and improved sheer load distribution as the screw degrades [4]. Moreover, bioabsorbable screws are radiolucent and do not require possible removal during revision. Theoretically, the design of bioabsorbable interference screws allows them to securely fixate the ACL graft to the bone tunnel throughout the healing process and then resorb through the body's natural pathways. Disadvantages have been reported, including decreased pullout strength and adverse bone resorption reactions leading to cyst formation [7].

Many varieties of bioabsorbable screws are available, each having different degradation characteristics depending on polymer choice and the specific implantation environment. Polylactic acid (PLA) screws have given excellent clinical results but contain highly variable degradation profiles; incomplete screw degradation has been noted after more than five years [6]. Polylactic‐co‐glycolic (PLG) screws have been shown to absorb fully within one year after implantation, leaving no evidence of bony replacement [8]. Osteoconductive materials have been developed to meet the need to replace of the degraded screw with bone. When mixed with calcium composites, PLA exhibits better evidence of bony ingrowth and replacement [5]. However, instances of foreign body reactions and tunnel widening are still present [8]. Tunnel widening may occur because of both mechanical and biological factors. Mechanical factors include accelerated rehabilitation and improper tunnel placement. Biological factors believed to be associated with tunnel widening include foreign body reactions or other responses that might preclude tendon–bone interface from healing.

**Figure 1:**
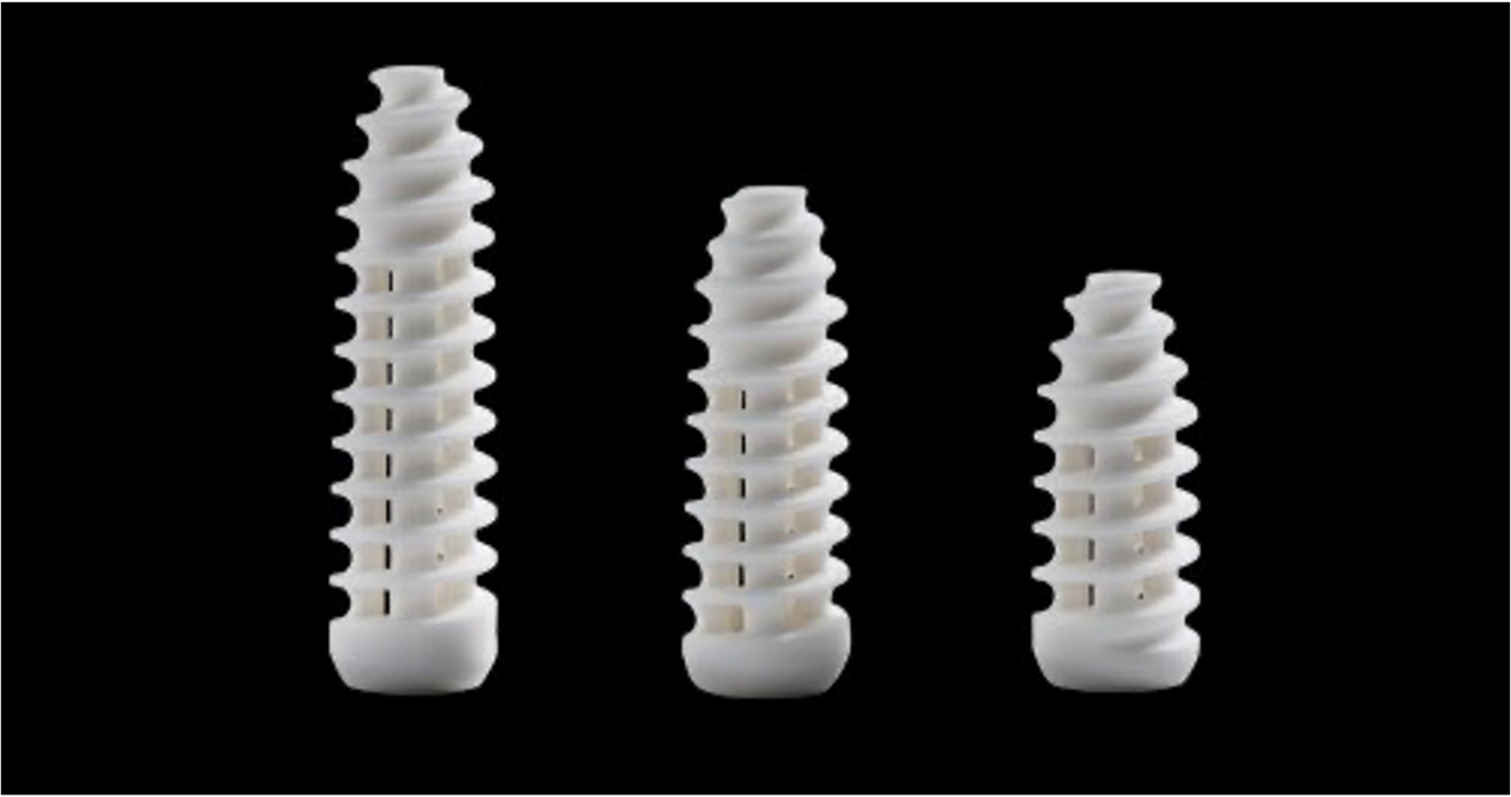
The Biosure Regenesorb interference screw with its fenestrated design provides circumferential communication with the tibial tunnel and is composed of an absorbable material consisting of a combination of calcium sulphate (20%), b‐TCP(15%) and PLGA (65%).

Smith & Nephew developed the Biosure Regenesorb interference screw as a novel approach to bioabsorbable interference screws. These screws are made from combined absorbable materials according to a fenestrated design that facilitates circumferential communication with the tibial tunnel (Figure 1). Absorbable material consists of a combination of calcium sulphate (20%), b‐Tricalcium phosphate (TCP) (15%), and polylactate gluconic acid (PLGA) (65%). Ovine animal studies have shown that the combination of calcium compounds and polymer results in 50% replacement with bone within a two‐year period [10]. The screw's open profile displaces less surface area than standard interference screws while maintaining comparable properties and fixation strengths. Furthermore, the Biosure Regenesorb interference screw's open design should permit bone ingrowth into the tunnel. No data are currently available on the Biosure Regenesorb interference screw's resorption characteristics and new bone formation abilities in the context of ACLR using soft tissue grafts in humans.

The present study's primary objective is to assess the tunnel geometry and screw resorption as evaluated by computer tomography (CT) scans performed in the immediate postoperative period and after one and two years for Biosure Regenesorb interference screws in the tibial tunnels of patients undergoing ACL reconstruction with soft tissue grafts. Knee stability and subjective knee function will also be evaluated.

## MATERIALS AND METHODS

### Study design

This study was a single‐centre prospective case series. Patients requiring ACL reconstruction following ACL injury would receive a soft tissue four‐strand hamstring graft ACLR using the Biosure Regenesorb interference screw for tibia fixation and were followed up for 24 months postoperatively. Men and women between the ages of 18 and 50 who presented with an unilaterally torn ACL as confirmed by functional testing and who met the eligibility criteria were recruited. A total of 12 subjects were enroled in the study. CT scan imaging evaluated screw resorption and bone tunnel structure at two weeks, 12 and 24 months. Patient‐reported outcomes and knee stability were evaluated at 12 months.

### Inclusion and exclusion criteria

#### Inclusion criteria

- An ACL tear requiring surgical reconstruction with semitendinosus and gracilis grafts.
- Willingness and ability to give voluntary informed consent to participate in the study.
- Willingness and ability. in the investigator's opinion, to cooperate with study procedures and willingness to return to the study site for all postoperative study visits.
- Age between 18 and 50 years at the time of surgery.
- ASA group 0–2 (limited medical illness).

#### Exclusion criteria

- Previous ACL reconstruction.
- Injury to posterior cruciate ligament, lateral collateral ligament and medial collateral ligament
- Displaced meniscus lesions and meniscus root lesions.
- Cartilage injury (ICRS Grade IV lesion > 2 cm^2^).
- Current malignant disease.
- Rheumatoid arthritis.
- Osteonecrosis, avascular necrosis or ankylosing spondylitis.
- Obesity (body mass index [BMI] > 35).
- Subject pregnant or intention to become pregnant during the study.
- Subject had received medical treatment within 6 weeks of enrolment with any of the following: glucocorticoids or growth hormone.
- Participation in another investigative trial.

#### The primary outcome

Bone and implant changes with respect to implant volume, tunnel volume and new bone formation in the originally drilled tibial tunnel as evaluated using quantitative CT scans after 1‐ and 2‐year follow‐up and compared to CT scans taken at two weeks postoperative.

#### Secondary outcomes

The secondary endpoints were as follows. Objective sagittal knee stability was evaluated as maximal sagittal knee translation measured using a KT‐1000 arthrometer (MEDmetric) and presented as side‐to‐side difference in mm [2]. Rotational stability was performed by Pivot shift test that was performed preoperatively and at postoperative follow‐up. Objective stability measures were performed by one experienced physiotherapist. Subjective outcome scores as evaluated by subjective International Knee Documentation Committee (IKDC) score ranging from 0 to 100 with 100 being the score representing completely normal knee function [11] as well as Knee Osteoarthritis Outcome score (KOOS) [15]. Sports‐related knee function was evaluated according to the Tegner activity scale ranging from 0 to 10, where 10 represents sports activity at a professional soccer level [17].

### Surgery

All patients underwent ACLR using an arthroscopically assisted technique. Gracilis and semitendinosus tendons were harvested through a small incision over the pes anserinus. All four strands were sutured separately with non‐resorbable sutures. The grafts were placed using an EndoButton CL implant (Smith & Nephew) for femoral fixation. The EndoButton loop length ensured that a minimum of 25 mm of the graft was placed in the femoral tunnel.

Femoral drilling was performed through an anteromedial portal, with the drill hole placed centrally in the femoral ACL footprint. A 55‐degree oblique tibial hole was drilled into the anterior tibial cortex between the anterior edge of the medial collateral ligament and the tibial tuberosity, ending in the centre of the ACL tibial footprint. The drill hole's diameter was sized according to the diameter of the four‐strand graft with half‐millimetre increments. The four‐strand graft was pulled into place. In all patients. tibial fixation was performed using the Biosure Regenesorb interference screw (Smith & Nephew) according to the diameter of the tibial drill hole (Figure 1). The screw was placed just inside the tibial cortex for optimal graft compression and fixation in the tibial tunnel. Signs of graft twisting or damage were visually inspected during screw positioning. The incision was closed in layers, and low‐suction drains were inserted into the joint and graft harvest area. The patients were discharged from the hospital 2–4 h postoperatively.

### Postoperative rehabilitation

Each patient's knee was allowed free range of motion from Day 1, followed by isometric quadriceps and passive flexion exercises. Patients were allowed full weight bearing, as tolerated by pain and effusion, with the aid of crutches for the first 2 postoperative weeks. Stationary bike exercises were used from the fourth postoperative week, and progressive quadriceps‐strengthening exercises were conducted from the sixth week. Running was permitted at 3 months postoperatively, followed by a return to lateral actions and contact sports at 12 months postoperatively or later. Rehabilitation was physiotherapist‐supervised for three months, and criterion‐based activity progression was applied.

### Outcome evaluation

#### CT

All CT examinations were performed on a single scanner (SomatomⓇ Definition FLASH with 128 detector rows; Siemens). Images were acquired through the knee using a standard protocol (FOV: 32 × 16 cm mA: 230 kVp: 120) with reconstructed 0.6‐mm slices in all planes on bone and soft tissue algorithms. Patients were imaged in the supine position with their feet loosely bound and held in a comfortable anterior orientation to reduce movement artifacts rather than to standardise their position.

##### Image analysis

**Figure 2:**
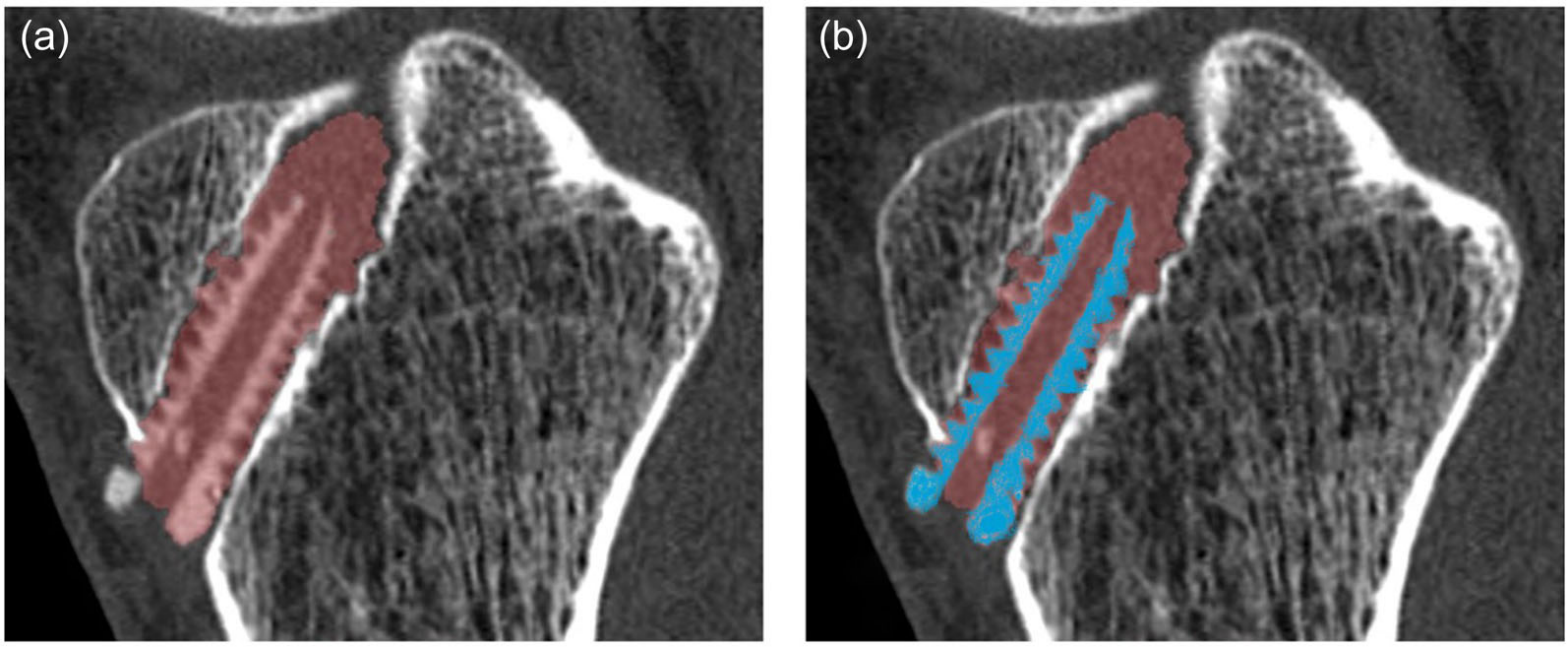
Examples of identification of the automated tunnel area (a) and screw area (b) in single sagittal reconstructed computed tomography sections.

Quantitative analysis of CT scans was performed using the Multi‐Modality Tumour Tracking (MMTT) software, available in the Intellispace Portal by Philips Healthcare (Koninklijke Philips N.V.). This software is specifically designed for segmenting specific volumes through signal thresholding and contour recognition (Figure 2), enabling precise measurements of even small, finely detailed elements. The segmentation process begins with a manual demarcation of the region of interest (e.g., screw material and total tunnel volume). The software then demarcates similar areas on consecutive slices, outlining the region of interest from surrounding tissue. The borders can be manually corrected to ensure the exact demarcation of the segmented areas and volumes. The volume and distribution of Hounsfield unit values are reported for each segmentation. Based on initial testing, the Hounsfield unit threshold for the Biosure Regenesorb screw material was established at 600. This enabled the software to identify the screw material as voxels within the drill tunnel with a Hounsfield unit value exceeding 600 (Figure 2b). The total tunnel volume was calculated by segmenting the entire volume, excluding both bone tissue and screw material (Figure 2a). Any adverse bone reactions such as tunnel widening and cyst formation was monitored by the changes in tunnel volume at the follow‐up times.

### Safety and adverse events

Adverse and serious adverse events were registered throughout the follow‐up period. Events that were monitored for were unexpected pain at tibial drill hole area, screw displacements, wound problems, infection and any reoperations.

Prior to enrolment, all subjects gave their informed consent. The study was approved by the local scientific ethical committee (Region Midtjylland Ethical Committee Approval no. 1‐10‐72‐145‐19) and by the Region Midtjylland Data Protection Agency and was conducted in accordance with the Helsinki Declaration.

### Statistical analysis

#### Data presentation and comparisons

Continuous variables were summarised according to the following summary statistics: number of observations, mean and standard deviations. For categorical variables median and minimum and maximum values.

CT scan data are presented as mean and standard deviations. A two‐tailed paired T‐test was used to comparing changes in screw and tunnel volumes from baseline to follow‐up and between follow‐up times. *p*‐Values of less than 0.05 were considered significant. All analyses were performed using STATA 16 version (StataCorp LP).

## RESULTS

### Baseline data

Table 1 presents the baseline epidemiological and patient‐reported outcome data. The mean age of 27 years, sex distribution, preoperative objective knee laxity of 3.4 mm and subjective knee function based on KOOS quality of life (QoL) score of 36 and Tegner activity scale scores of 4 are levels that are characteristic for ACL‐injured patients. As such the investigated cohort may be deemed representative of ACL patients in general.

**Table 1:** Demographic and patient‐reported outcome data at baseline.

Patients	*N* = 12
Gender (M/F)	8/4
Age (mean [range])	27.2 (21–48)
BMI (mean [range])	24.5 (20–33)
KT‐1000 side‐to‐side difference in Lachman laxity mm (mean [SD])	3.4 (1.4)
KOOS (mean [SD])	
‐ Symptoms	66 (21)
‐ Pain	70 (15)
‐ ADL	82 (15)
‐ Sport	37 (26)
‐ QoL	36 (16)
IKDC (mean [SD])	51 (18)
Tegner Activity Scale (mean [ange])	4 (3–8)

Abbreviations: BMI, body mass index; IKDC, International Knee Documentation Committee; KOOS, Knee Osteoarthritis and Injury Outcome Score; QoL, quality of life; SD, standard deviation.

### CT scanning results

#### One year

**Figure 3:**
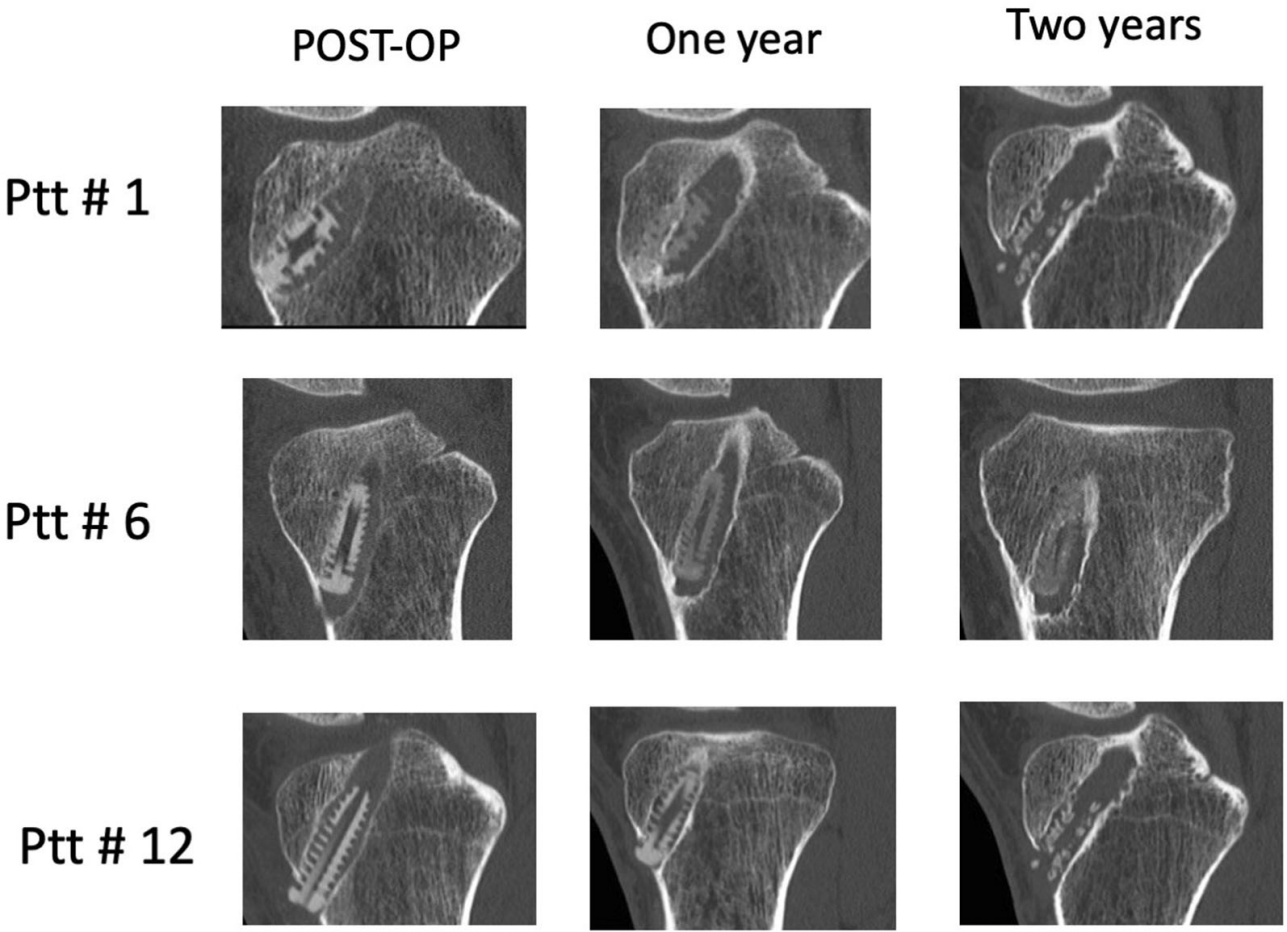
Three examples of the screw resorption process with representative computed tomography scan images in the immediate postoperative period and at the one‐ and two‐year follow‐ups.

The CT scanning analyses investigated the development of Biosure Regenesorb screw resorption and the volume of the tibial bone tunnel in which the screw was inserted to fix the soft tissue ACL graft (Table 2). The screw resorption screw volume changed from 0.63 cm^3^ immediately postoperative to 0.30 cm^3^ at the 1‐year follow‐up visit (*p* < 0.01). Figure 3 presents a visual impression of the resorption development in three representative cases.

**Table 2:** Computed tomography scanning data for all 12 patients with a presentation of screw volume tunnel volume and new bone formation at immediate postoperative, 1 year and 2 years follow‐up.

Patient	Tunnel diameter at surgery (mm)	Screw diameter (mm)	Baseline screw volume (cm^3^)	Baseline total tunnel volume (cm^3^)	1 Year screw volume (cm^3^)	1 Year total tunnel volume (cm^3^)	2 Year screw volume (cm^3^)	2 Year total tunnel volume (cm^3^)	New bone volume in the screw
1	7.50	8.00	0.56	2.41	0.36	3.16	0.33	2.87	‐
2	9.00	9.00	0.63	2.91	0.19	3.54	0.24	3.09	0.05
3	8.00	8.00	0.67	3.57	0.27	3.42	0.39	3.49	0.12
4	9.00	9.00	0.69	2.93	0.39	3.15	0.38	3.06	‐
5	8.00	8.00	0.78	3.80	0.53	5.00	0.24	3.71	‐
6	8.50	9.00	0.45	4.46	0.17	3.24	0.17	3.24	‐
7	7.50	8.00	0.59	2.87	0.29	2.87	0.29	2.86	‐
8	8.50	9.00	0.65	3.56	0.10	2.42	0.17	3.27	0.07
9	8.00	8.00	0.52	2.04	0.17	2.35	0.37	2.66	0.20
10	8.00	8.00	0.68	3.09	0.38	3.18	0.21	2.84	0.05
11	8.50	9.00	0.73	4.02	0.39	3.38	0.44	4.34	‐
12	8.50	9.00	0.67	2.22	0.32	2.57	0.16	2.34	‐
**Mean**			**0.63**	**3.16**	**0.30**	**3.19**	**0.28**	**3.15**	**0.10**

Tibial tunnel volume remained practically unchanged after the first year, with a volume of 3.16 cm^3^ immediately postoperative to 3.19 cm^3^ at the 1‐year follow‐up visit.

New bone formation within the Biosure Regenesorb interference screw threads and centre tunnel was observed in very minor amounts (<1% of tunnel volume) in some patients. These small new bone formations were not quantifiable.

#### Two years

Between the first and second years postoperatively. the screws showed further resorption from 0.30 cm^3^ to 0.28 cm^3^ on average. This represents screw resorption to 44% of the original screw material volume at 2 years. Total tibial tunnel volume was unchanged from the first to the second year with a volume of 3.19 cm^3^ at 1 year to 3.15 cm^3^ at the 2‐year follow‐up. Whereas very limited new bone formation in the areas of screw and tunnels was observed at the 1‐year follow‐up. At the 2‐year follow‐up. five out of the 12 screw areas exhibited increased bone content. Representing approximately 3% of tunnel volume at 2 years.

### One‐year clinical follow‐up data

Table 3 presents the 1‐year follow‐up knee stability and patient‐reported outcome data. Knee stability was found to be significantly improved with a side‐to‐side sagittal knee laxity of 0.9 mm (*p* < 0.05). A positive Lachman and pivot shift finding was observed in only one patient. For the subjective outcomes of KOOS the most responsive subscales of sport activity and quality of life improved significantly with 26 and 25 points, respectively (*p* < 0.05), which more than the minimal clinically relevant change of 26 and 25 points, respectively. The subjective IKDC score showed an improvement of 14 points (*p* < 0.05), which is also significant and more than the minimal clinically relevant change for the IKDC score.

**Table 3:** One‐year follow‐up clinical outcome data.

Patients *N* = 12	Baseline	One year
Rolimeter (mean [SD])	3.4 (1.4)	0.9 (1.4)*
Positive Lachmann (*n* [%])	12 (100)	1 (8)
Positive Pivot Shift (*n* [%])	10 (83)	1 (8)
IKDC Obj		
‐ Grade A (*n* [%])	1 (8)	5 (42)
‐ Grade B (*n* [%])	4 (33)	7 (58)
‐ Grade C (*n* [%])	7 (59)	0 (0)
KOOS (mean [SD])		
‐ Symptoms	66 (21)	70 (22)*
‐ Pain	70 (15)	81 (13)*
‐ ADL	82 (15)	90 (12)*
‐ Sport	37 (26)	59 (51)*
‐ QoL	36 (16)	52 (14)*
Subj. IKDC score (mean [SD])	51 (18)	65 (15)*
Tegner activity scale (mean [range])	4 (3–8)	4 (1–5)

*Note*: Significant change from preoperative to follow‐up (*p* < 0.05).

Abbreviations: IKDC, International Knee Documentation Committee; KOOS, Knee Osteoarthritis and Injury Outcome Score; QoL, quality of life; SD, standard deviation.

### Adverse events

No adverse events were recorded for the 12 included patients during the two‐year follow‐up period.

## DISCUSSION

The present study's primary finding was that a novel biocomposite interference screw composed of a combination of calcium sulphate, b‐TCP and PLGA with open architecture used in the tibial tunnel of subjects undergoing ACL reconstruction with soft tissue grafts resorbed to 50% within the first year with further resorption in the second year without causing adverse bone reactions, such as tunnel widening or cyst formation.

Another important finding was that only limited new bone formation in association with the resorption of the screw was found. By comparison, one animal study investigated bone ingrowth and fixation strength after 6 months of perforated PLGA screws in a sheep model and found bone ingrowth in the perforations and a tendency to increase fixation strength of the perforated screw compared to non‐perforated screws [10]. In a clinical study aiming to investigate the screw resorption of poly‐DL‐Lactide (PDLLA) screws with perforations to accelerate its resorption. But bone ingrowth was not investigated in this study. The screw exhibited complete resorption after 30 months [1]. Another study investigated long‐term screw remodelling and bone ingrowth in a composite hydroxyapatite (HA)‐polylactide (HA‐PLLA) screw area. The study found that the HA‐PLLA material stimulated osteoconductivity. Resulting in almost complete screw bone ingrowth after five years with 30% ingrowth after 1 year [12]. The higher bone formation associated with HA‐PLLA screws compared to the present study may be attributable to the different calcium phosphate/polymer composition with the HA component potentially being more osteoconductive than the tricalcium phosphate component of the Biosure Regenesorb screw.

The present study's secondary objectives were to assess changes in IKDC and KOOS and Tegner activity scale scores from baseline to 12 months postoperatively. At the 12‐month follow‐up visit a statistically significant improvement from baseline was observed. The IKDC score at 12 months was 65, which represented a 14‐point increase from baseline that was clinically relevant. However, a follow‐up score of 65 is lower than the average IKDC levels found in large‐volume registry ACLR outcome studies [14, 16]. Another secondary outcome was knee stability, which was found to improve significantly from a preoperative sagittal laxity of 3.7 mm to a 12‐month postoperative laxity of 0.9 mm. The Tegner activity scale did not improve from a score of 4 at baseline and at the 1‐year follow‐up. The likely reason for this is that the patients had a high baseline score of 4, where the normal score before ACL injury treatment is 3. Moreover, the Tegner score only improves after returning to more impact‐intensive sports activities and for these activities the 1‐year follow‐up is still somewhat early.

Another secondary outcome was knee stability, which was found to improve significantly from a preoperative sagittal laxity of 3.7 mm to a 12‐month‐postoperative laxity of 0.9 mm.

No adverse events were noted in relation to the use of the open architecture resorbable interference screw during hamstring ACL reconstruction. Significantly, no graft damage or twisting events were observed during screw positioning.

Several imaging modalities have been recommended for the visualisation of bone remodelling and tunnel widening in the tibial tunnel including x‐ray, computed tomography (CT), and magnetic resonance imaging (MRI). Owing to concerns regarding patients' exposure to radiation, many ACLR studies have used MRI for imaging endpoints. Several other studies have used MRI to evaluate screw resorption and bone formation after the use of a resorbable interference screw. While MRI scanning may be sufficient to identify screw remnants, its ability to identify newly formed calcified tissue is poor [1, 13]. However, a study has shown that the appearance of bone resorption on MRI does not necessarily correlate with the actual processes occurring in the bone [3]. CT scannings high‐resolution capabilities allow for quantitatively assess bone ingrowth as masks created in different areas of the bone tunnel (e.g., interference screws, grafts and tunnels) may be applied at baseline and compared at subsequent timepoints [18]. CT scanning is the gold standard for assessing bone healing and formation [8]. A strength of the study was thus that high‐resolution CT scanning was used to evaluate implant resorption and new bone formation and bone ingrowth.

The present study's clinical relevance lies in the fact that open architecture resorbable interference screws used for tibia fixation in soft tissue ACL reconstruction resorb gradually over 2 years without causing any tunnel widening. These properties may offer an advantage in revision surgery situations in which resorbed implants and maintained tunnel sizes might result in reusable tunnels without the need for implant removal.

The present study has some limitations. With only 12 patients included. the design was sufficient to describe the desired biological phenomenon of implant resorption and bone formations but lacked the ability to generate complete data for clinical outcomes such as knee stability, subjective patient‐reported outcomes and failure rates. but the clinical outcome data were primarily included to identify adverse events. Another limitation is that no control group was included.

## CONCLUSION

The Biosure Regenesorb interference screw used for tibial soft tissue graft fixation during ACL reconstruction demonstrates rapid resorption to half screw volume after one year with further resorption to 44% of total screw material without affecting tibial tunnel size after two years. Screw resorption did not result in any adverse tunnel widening. New bone formation within the Biosure Regenesorb screw was observed to a limited degree at two years follow‐up.

## AUTHOR CONTRIBUTIONS

Martin Lind: Article writing, data analysis, patient surgery and follow‐up. Torsten Nielsen: Data analysis, patient surgery and follow‐up. Ole Gade Sørensen: Article writing, patient surgery and follow‐up. Bjarne Mygind‐Klavsen: Article writing, patient surgery and follow‐up. Peter Faunø: Article writing, patient surgery and follow‐up. Flemming Kromann Nielsen: CT data analysis.

## CONFLICT OF INTEREST STATEMENT

The research presented here was supported by a research grant from Smith & Nephew. The main author, ML, served as a scientific consultant during the manuscript's preparation. ML has also provided educational services for Smith & Nephew. The other authors declare no conflict of interest.

## ETHICS STATEMENT

The study was approved by the local scientific ethical committee (Region Midtjylland Ethical Committee Approval no. 1‐10‐72‐145‐19) and by the Region Midtjylland Data Protection Agency and was conducted in accordance with the Helsinki Declaration.

## Data Availability

The data sets used and/or analysed during the current study are available from the corresponding author on reasonable request.
